# Production of recombinant cholesterol oxidase containing covalently bound FAD in *Escherichia coli*

**DOI:** 10.1186/1472-6750-10-33

**Published:** 2010-04-21

**Authors:** Federica Volontè, Loredano Pollegioni, Gianluca Molla, Luca Frattini, Flavia Marinelli, Luciano Piubelli

**Affiliations:** 1 Dipartimento di Biotecnologie e Scienze Molecolari, Università degli Studi dell'Insubria via J.H. Dunant 3, 21100 Varese, Italy; 2 Centro Interuniversitario di Ricerca in Biotecnologie Proteiche "The Protein Factory", Politecnico di Milano and Università degli Studi dell'Insubria, Varese, Italy

## Abstract

**Background:**

Cholesterol oxidase is an alcohol dehydrogenase/oxidase flavoprotein that catalyzes the dehydrogenation of C(3)-OH of cholesterol. It has two major biotechnological applications, i.e. in the determination of serum (and food) cholesterol levels and as biocatalyst providing valuable intermediates for industrial steroid drug production. Cholesterol oxidases of type I are those containing the FAD cofactor tightly but not covalently bound to the protein moiety, whereas type II members contain covalently bound FAD. This is the first report on the over-expression in *Escherichia coli *of type II cholesterol oxidase from *Brevibacterium sterolicum *(BCO).

**Results:**

Design of the plasmid construct encoding the mature BCO, optimization of medium composition and identification of the best cultivation/induction conditions for growing and expressing the active protein in recombinant *E. coli *cells, concurred to achieve a valuable improvement: BCO volumetric productivity was increased from ~500 up to ~25000 U/L and its crude extract specific activity from 0.5 up to 7.0 U/mg protein. Interestingly, under optimal expression conditions, nearly 55% of the soluble recombinant BCO is produced as covalently FAD bound form, whereas the protein containing non-covalently bound FAD is preferentially accumulated in insoluble inclusion bodies.

**Conclusions:**

Comparison of our results with those published on non-covalent (type I) COs expressed in recombinant form (either in *E. coli *or *Streptomyces *spp.), shows that the fully active type II BCO can be produced in *E. coli *at valuable expression levels. The improved over-production of the FAD-bound cholesterol oxidase will support its development as a novel biotool to be exploited in biotechnological applications.

## Background

Cholesterol oxidase (EC 1.1.3.6, CO) is an alcohol dehydrogenase/oxidase flavoprotein that catalyzes the dehydrogenation of C(3)-OH of a cholestane system to yield the corresponding carbonyl product. During the reductive half-reaction, the oxidized FAD cofactor accepts a hydride from the substrate and in the ensuing oxidative half-reaction, the reduced flavin transfers the redox equivalents to molecular oxygen yielding hydrogen peroxide. Interestingly, CO also catalyzes the isomerization of cholest-5-en-3-one (the product of the redox reaction) to cholest-4-en-3-one.

CO is an useful biotechnological tool employed: i) for the determination of serum (and food) cholesterol levels, ii) as biocatalyst providing valuable intermediates for industrial steroid drug production, iii) as larvicidal protein (the one from *Streptomyces *sp. strain A19249) that has being developed as an insecticide against *Coeloptera*. Furthermore, CO has been proposed as a virulence factor playing a role in infections caused by the pathogen bacterium *Rhodococcus equi*. Since this enzymatic activity is unique to bacteria, it may represent a novel molecular target for antibiotic discovery. CO also acts as signaling protein for the biosynthesis of polyene macrolide pimaricin in *Streptomyces natalensis*. For a review see [1-4].

**Figure 1:**
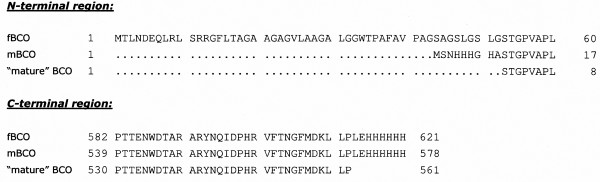
**Sequence alignment of the BCO forms expressed in *E. coli***. fBCO: Protein obtained by subcloning the fBCO-cDNA into pET24b(+) using the *Nde*I/*Xho*I restriction sites; mBCO: protein obtained by subcloning the mBCO-cDNA into pET24b(+) using the *Nde*I/*Xho*I restriction sites. For comparison, the sequence of the "mature" form of BCO (as determined from the 3-D structure, PDB code 1I19) is reported.

CO is produced, and frequently secreted, by a variety of prokaryotic microorganisms. CO from *Brevibacterium sterolicum *(BCO) is produced as a 613 amino acid long precursor (full length BCO, fBCO) that lacks the N-terminal pre-sequence (52 amino acid long) to yield the mature and fully active enzyme form (Figure 1). BCO is the prototype of type II COs: it contains a FAD cofactor covalently bound to His69 and belongs to the family of vanillyl-alcohol oxidase, whose members possess a fold proposed to favour covalent flavinylation [5]. The members of CO class type I (such as the one from *Streptomyces hygroscopicus *or *Rhodococcus equi*) contain the FAD cofactor tightly but non-covalently bound to the protein moiety. Enzymes belonging to these two classes show no significant sequence identity, possess different tertiary structure, and have different kinetics mechanism and redox potentials [2,3]. In recent years, we elucidated the function of covalent flavin leakage studying the H69A BCO mutant, in which the amino acid substitution prevents formation of the histidyl-FAD bond. The mutant BCO shows a similar structure compared to the wild-type enzyme and retains catalytic activity (both the oxidation and the isomerisation reaction) but with a 35-fold decrease in turnover number, a 100 mV more negative midpoint reduction potential, and a significant lower stability to denaturant agents and temperature than the covalent counterpart [6-8]. We thus concluded that this covalent bond represents a structural device to both modify the reduction potentials (catalytic power) of the cofactor and to stabilize the tertiary structure.

BCO shows peculiar characteristics that makes it a suitable biotechnological tool, such as a higher apparent k_cat _value on cholesterol as compared to non-covalent CO (2-15 fold higher, depending on the experimental conditions used) and a 5-fold lower K_m,O2 _[2,9]. Furthermore, the stable cofactor interaction makes BCO a better choice than non-covalent COs for those applications where FAD dissociation reduces the biocatalyst performances (e.g., immobilized-based processes and biosensors) [3]. In fact, the apoprotein of *S. hygroscopicus *CO (type I-CO) is highly unstable [2].

Despite its biotechnological importance, no detailed analysis has been so far reported concerning the over-production of type II-CO members (containing covalently bound FAD) as recombinant proteins. Previous results revealed that recombinant wild-type BCO (as well as some of its point mutants) were produced as a mixture of two enzyme forms containing covalently (flavinylated) and non-covalently bound FAD, as judged by spectral and kinetics analysis [10]. This is the first report of BCO over-expressed in *E. coli *using different constructs and media, and under cultivation/induction controlled conditions at flask and fermentor scale. The aim of this work is to verify the effect of these factors on the overall BCO production as well as on its partitioning between the form containing covalently-bound FAD (the fully active enzyme species) and its non-covalent (marginally active) counterpart. The promising results hereby reported, will favour the use of this recombinant strain for future CO biotechnological production.

## Results

### Expression of different BCO forms

Synthetic cDNAs encoding full length and mature BCO forms (whose codon usage was optimized for *E. coli *expression) were subcloned into pET24b(+) plasmid giving a 621 amino acid long protein (fBCO, of 67.51 kDa) or a 578 amino acid long BCO (mBCO, of 63.59 kDa), respectively, both containing a C-terminal His tag (Figure 1). The mBCO form also contains an additional 9-residues long N-terminal sequence, previously demonstrated to be necessary for successful protein crystallization [2,8]. In preliminary experiments both the constructs were used to express BCO in *E. coli *BL21(DE3)pLysS cells grown in LB medium, by inducing the protein expression with 1 mM IPTG at different times of growth and collecting the cells (grown at 25 or 37°C) 3, 7 or 18 hours after induction. As summarized in Table 1, the mBCO form shows the highest expression of BCO activity. In the cases of fBCO, the best expression conditions (260 U/L culture) are obtained when IPTG is added during exponential growth phase and cells are collected after a prolonged (overnight) incubation at 25°C. In the same conditions, a volumetric productivity of nearly 500 U/L culture of mBCO with a specific activity of 0.54 U/mg protein is obtained. When induction is performed in the stationary phase at 25°C slightly lower expression levels of mBCO (400 U/L culture) is achievable after three hours, indicating that a more rapid expression kinetics occurs in these conditions.

**Table 1:** Expression of the BCO forms using different growth and induction conditions.

Phase of induction	Plasmid (BCO form)	Time after induction (h)	Temperature after induction			
			
			25°C		37°C	
			
			U/mg_protein_	U/L_culture_	U/mg_protein_	U/L_culture_
Exponential phase (OD_600 nm_~1)	pET-fBCO (fBCO)	0	b.d.	b.d.	b.d.	b.d.
		3	≤ 0.01	< 1.0	≤ 0.01	< 1.0
		7	0.07 ± 0.005	2.0 ± 0.3	0.10 ± 0.05	< 1.0
		18	0.16 ± 0.05	260 ± 80	0.12 ± 0.02	1.8 ± 0.2
	
	pET-mBCO (mBCO)	0	b.d.	b.d.	b.d.	b.d.
		3	0.31 ± 0.02	130 ± 10	0.09 ± 0.02	50 ± 11
		7	0.38 ± 0.14	190 ± 50	1.0 ± 0.3	390 ± 45
		18	0.54 ± 0.15	500 ± 120	0.17 ± 0.06	240 ± 20

Stationary phase (OD_600 nm_~2)	pET-fBCO (fBCO)	0	b.d.	b.d.	b.d.	b.d.
		3	≤ 0.01	< 1.0	≤ 0.01	< 1.0
		7	≤ 0.01	1.1 ± 0.1	≤ 0.01	< 1.0
		18	0.03 ± 0.015	2.5 ± 0.4	0.02 ± 0.01	1.8 ± 0.2
	
	pET-mBCO (mBCO)	0	b.d.	b.d.	b.d.	b.d.
		3	0.52 ± 0.12	400 ± 10	0.22 ± 0.06	150 ± 35
		7	0.28 ± 0.02	180 ± 20	0.55 ± 0.18	180 ± 10
		18	0.13 ± 0.02	110 ± 30	0.12 ± 0.05	85 ± 9

In all cases, *E. coli *cells carrying the recombinant pET24b(+)-BCO plasmids were cultivated in LB medium and protein expression was induced by 1 mM IPTG addition. b.d., below detection limit. Data are expressed as mean ± S.D., n = 3.

SDS-PAGE Analysis of soluble and insoluble fractions (pellet) after lyses of cells induced with 1 mM IPTG at an OD_600 nm_~1, clearly indicates that at the post-induction temperature of 37°C a large amount of the fBCO as well as mBCO protein is produced in the insoluble fraction (not shown). When cells are incubated at 25°C after the induction, a significant amount of recombinant fBCO is found in the insoluble fraction, whereas mBCO is expressed to a large extent in the soluble form. Based on these results, the subsequent investigations were focused on the mBCO form.

### Optimization of the mBCO production conditions

The expression of recombinant mBCO was then investigated using a modified 2.6-kb-shorter version of pET24Δ plasmid as expression vector (i.e., pET24Δ plasmid, see Methods for details), that was previously reported to increase protein expression yield [11]. In fact, the expression level in LB broth under experimental conditions similar to those reported above (induction at the exponential growth phase by 1 mM IPTG followed by overnight incubation at 25°C) is about twice than that observed with the longer plasmid: 860 U/L with a specific activity of 1.7 U/mg protein are thus achieved (Table 2).

**Table 2:** Effect of medium composition, IPTG concentration and time of protein induction on mBCO expression.

Medium	[IPTG] (mM)	Time after induction (h)	Biomass (g/L_culture_)	Specific activity (U/mg_protein_)	Volumetric productivity (U/L_culture_)
LB	/	0	5.9 ± 0.3	< 0.01	0.5 ± 0.3
	0.25	3	5.9 ± 0.8	1.5 ± 0.4	550 ± 100
		18	7.3 ± 0.4	1.5 ± 0.2	890 ± 280
	0.5	3	6.1 ± 0.5	1.4 ± 0.2	460 ± 170
		18	5.9 ± 0.2	1.4 ± 0.4	700 ± 220
	1	3	5.9 ± 0.8	1.5 ± 0.1	570 ± 160
		18	6.8 ± 0.1	1.7 ± 0.6	860 ± 190

TB	/	0	7.6 ± 0.9	≤ 0.02	8.9 ± 2.3
	0.25	3	10.8 ± 1.4	1.1 ± 0.2	830 ± 50
		18	26.2 ± 3.9	5.9 ± 1.3	13300 ± 350
	0.5	3	10.6 ± 1.4	1.0 ± 0.2	1000 ± 200
		18	24.8 ± 4.0	3.3 ± 0.5	11400 ± 1800
	1	3	10.3 ± 0.8	1.2 ± 0.1	940 ± 50
		18	24.3 ± 2.3	3.9 ± 0.1	11100 ± 1700

TB/glucose	/	0	8.5 ± 1	≤ 0.02	3.6 ± 1.3
	0.25	3	14.9 ± 1.9	3.2 ± 0.6	1260 ± 80
		18	21.0 ± 3.1	3.1 ± 0.7	5440 ± 140
	0.5	3	15.9 ± 2.1	2.3 ± 0.5	1220 ± 240
		18	20.0 ± 3.2	5.1 ± 0.8	6400 ± 900
	1	3	17.4 ± 1.4	2.4 ± 0.2	1340 ± 70
		18	20.4 ± 1.9	5.3 ± 0.1	6300 ± 900

SB	/	0	11.4 ± 1.3	≤ 0.01	15 ± 8
	0.25	3	20.2 ± 2.6	3.3 ± 0.7	4050 ± 240
		18	16.0 ± 2.4	3.2 ± 0.7	8200 ± 220
	0.5	3	22.0 ± 2.4	1.3 ± 0.7	3160 ± 690
		18	23.2 ± 0.8	2.1 ± 0.3	9500 ± 2100
	1	3	18.9 ± 2.4	1.8 ± 0.4	2520 ± 150
		18	18.8 ± 2.8	4.1 ± 0.9	7900 ± 210

*E. coli *cells carrying the recombinant pET24Δ-mBCO plasmid were grown at 37°C until OD_600 nm_~1.5-2, added of IPTG, incubated at 25°C and harvested after 3 or 18 hour from the protein induction. Data are expressed as mean ± S.D., n = 3.

In order to optimize mBCO expression, several experimental parameters were modified at flask-scale: cultivation medium composition, IPTG concentration, growth phase at induction, and incubation temperature after induction. The medium composition significantly affects mBCO expression. Very low mBCO expression is observed in M9 minimal medium (0.7 U/L and 0.003 U/mg protein). As expected, richer media support higher biomass production: more than 20 g cells/L are produced in TB, in TB/glucose (a TB medium containing 5 g/L glucose instead of glycerol) and SB media following overnight growth after induction, in comparison to the 6-7 g/L cells sustained by LB medium (Table 2). The medium composition also appreciably affects the mBCO's volumetric productivity and specific activity in the crude extract: these figures are significantly higher in richer media with respect to LB. The presence of glycerol as carbon source (TB medium) seems to play a positive effect on mBCO production (up to 13300 U/L culture, 5.9 U/mg protein), whereas when it is replaced by glucose (TB/glucose medium) the volumetric yield is halved (see below and Table 2). SB medium sustains a more rapid production of biomass (more than 20 g/L cells achieved in 3 hours after IPTG addition) in comparison to TB, but this is not accompanied by an increase in mBCO productivity, which did not exceed the 9500 U/L culture and the 2.1 U/mg protein. In all the tested media, mBCO production is higher when the cells are collected 18 hours after induction. The concentration of IPTG does not appear to exert a significant effect either on cell growth or on protein expression: similar values of biomass and mBCO expression are obtained at ≥ 0.25 mM inducer concentration (Table 2). This is a valuable result since IPTG represents the most expensive component used in *E. coli *recombinant protein production.

In richer media, such as TB and SB, induction of mBCO expression during mid-exponential phase (OD_600 nm _= 1.5-2) results in higher production than at early exponential phase (OD_600 nm _= 0.8-1): more than 11000 vs. 6000 U/L culture with a specific activity of 3.9 vs. 2.8 U/mg protein are produced in TB following IPTG addition at and OD_600 nm_~2 and ~1, respectively, in comparison to 7800 vs. 4800 U/L culture and 4.0 vs. 2.4 U/mg protein in SB medium. Reduced expression of mBCO is indeed observed following addition of IPTG to cells grown in TB from the late exponential phase (OD_600 nm_~6-7) till the advanced stationary phase (OD_600 nm_~9) and collected after 24 hours (data not shown).

Concerning the impact of medium components, glycerol appears to play a major role on mBCO expression, whereas phosphate buffer seems to exert a minor effect. Addition of glycerol to SB medium increases both the specific and the volumetric productivity, whereas addition of phosphate salts does not (Table 3). A similar result is observed in TB medium: removal of phosphate salts does not negatively influence the biomass production or the mBCO expression, whereas in the absence of glycerol both these parameters are significantly reduced (Table 3). Interestingly, glycerol addition to LB medium does not change the volumetric productivity (Table 3). Indeed, increasing the amount of glycerol in TB medium up to 20 mL/L generates an improvement in the volumetric productivity as well as in the specific activity of mBCO, but it does not seem to significantly affect cell growth (Table 3). At 20 mL/L of glycerol, about 14000 U/L culture and 5.9 U/mg protein are produced. The temperature of incubation after IPTG addition is confirmed as a crucial factor affecting mBCO expression: in the four different tested media, the highest enzyme production is observed at 25°C and at 18°C, while incubation at 37°C results in a dramatic activity decrease (Table 3) and at 42°C cell lysis is observed. Under all conditions tested, no mBCO activity is detected in the cultivation medium.

**Table 3:** Effect of glycerol, phosphate and incubation temperature after IPTG addition on mBCO expression.

Medium	Temperature after induction (°C)	Biomass (g/L_culture_)	Specific activity (U/mg_protein_)	Volumetric productivity (U/L_culture_)
LB	18	8.7 ± 1.9	1.3 ± 0.3	770 ± 130
	25	6.8 ± 0.7	1.7 ± 0.3	860 ± 80
	37	5.3 ± 0.5	≤ 0.01	3.0 ± 0.3

LB+G	25	12.7 ± 1.3	0.87 ± 0.10	760 ± 80
	37	10.8 ± 1.1	0.08 ± 0.01	65 ± 7

TB	18	18.7 ± 3.3	3.8 ± 0.6	8030 ± 1400
	25	24.3 ± 2.6	3.9 ± 1.0	11100 ± 1100
	37	22.7 ± 2.4	≤ 0.01	9.0 ± 0.9

TB-Pi	25	28.3 ± 3.1	4.1 ± 1.1	13100 ± 1300

TB-G	25	18.0 ± 1.0	3.0 ± 0.5	6140 ± 200
	37	6.0 ± 0.3	≤ 0.01	7.0 ± 0.2

TB+4G	25	22.4 ± 2.5	2.8 ± 0.5	11000 ± 400

TB+G	25	24.3 ± 2.6	3.9 ± 0.9	11200 ± 1100

TB+20G	25	21.9 ± 1.5	5.9 ± 1.0	14200 ± 2800

TB-Pi-G	25	19.8 ± 1.1	2.8 ± 0.5	8000 ± 260

SB	25	18.8 ± 0.7	4.1 ± 0.2	7900 ± 140

SB+Pi	25	19.5 ± 1.4	3.5 ± 1.1	8720 ± 2340

SB+G	25	22.5 ± 1.6	4.6 ± 0.8	11100 ± 1500

SB+G+Pi	25	19.2 ± 1.3	5 ± 1	10300 ± 1700

*E. coli *cells carrying the recombinant pET24Δ-mBCO plasmid were grown at 37°C, BCO expression was induced by 1 mM IPTG at OD_600 nm _= 1.5-2 and harvested after 18 hours at different temperatures. G: glycerol. +G, +Pi: media added of glycerol (8 mL/L) and/or phosphate salts (70 mM), respectively; +4G and +20G: media added of 4 mL/L and 20 mL/L glycerol, respectively; -G, -Pi: media deprived of glycerol and/or phosphate salts, respectively. Data are expressed as mean ± S.D., n = 3.

### Fermentor scaling up

**Figure 2:**
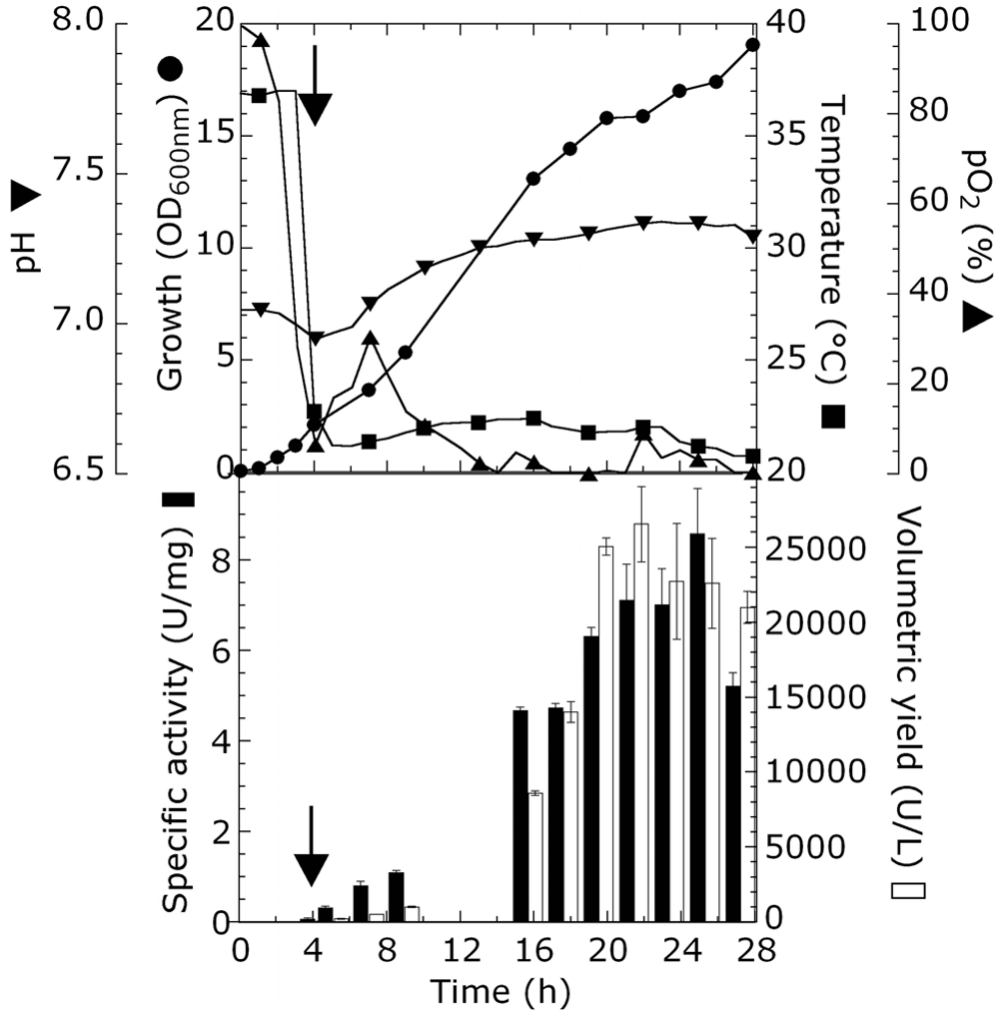
**Production of mBCO in 2 L-batch fermentation of recombinant *E. coli *cells under optimized conditions**. Top panel: time course of pH (down triangle), pO_2 _(up triangle), temperature (square), and growth curve measured as OD_600 nm _(circle). Bottom panel: production of mBCO measured as specific activity (U/mg, filled bars) and volumetric productivity (U/L, empty bars). The arrow indicates the moment of IPTG addition.

The best conditions set up at flask level were scaled up at 2 L fermentor scale. Cell were grown at 37°C in TB medium added with 20 mL/L glycerol and IPTG-induced during early-exponential phase (OD_600 nm _= 1.5-2). At the time of induction, temperature was shifted to 21°C, and the kinetics of BCO production in this condition was followed for additional 24 hours (Figure 2). After a short lag phase of one-two hours, the culture starts to grow exponentially: 25 g cells/L are produced in 18-20 hours after the inoculum. More than 25000 U/L culture and 7 U/mg BCO are accumulated by cells entering into stationary phase (18-20 hours). After 24 hours, protein production starts to slowly decrease: comparison by means of Western blot and activity assays (see below) of the amount of expressed BCO indicates that the observed decrease is mainly due to protein degradation (i.e. a similar decline in terms of BCO production and enzyme activity is observed). Indeed, the amount of BCO activity recovered from fermentation broth is <2% of the intracellular enzymatic activity, confirming that the protein is not released from the cells.

### Production of covalent vs. non-covalent mBCO

**Figure 3:**
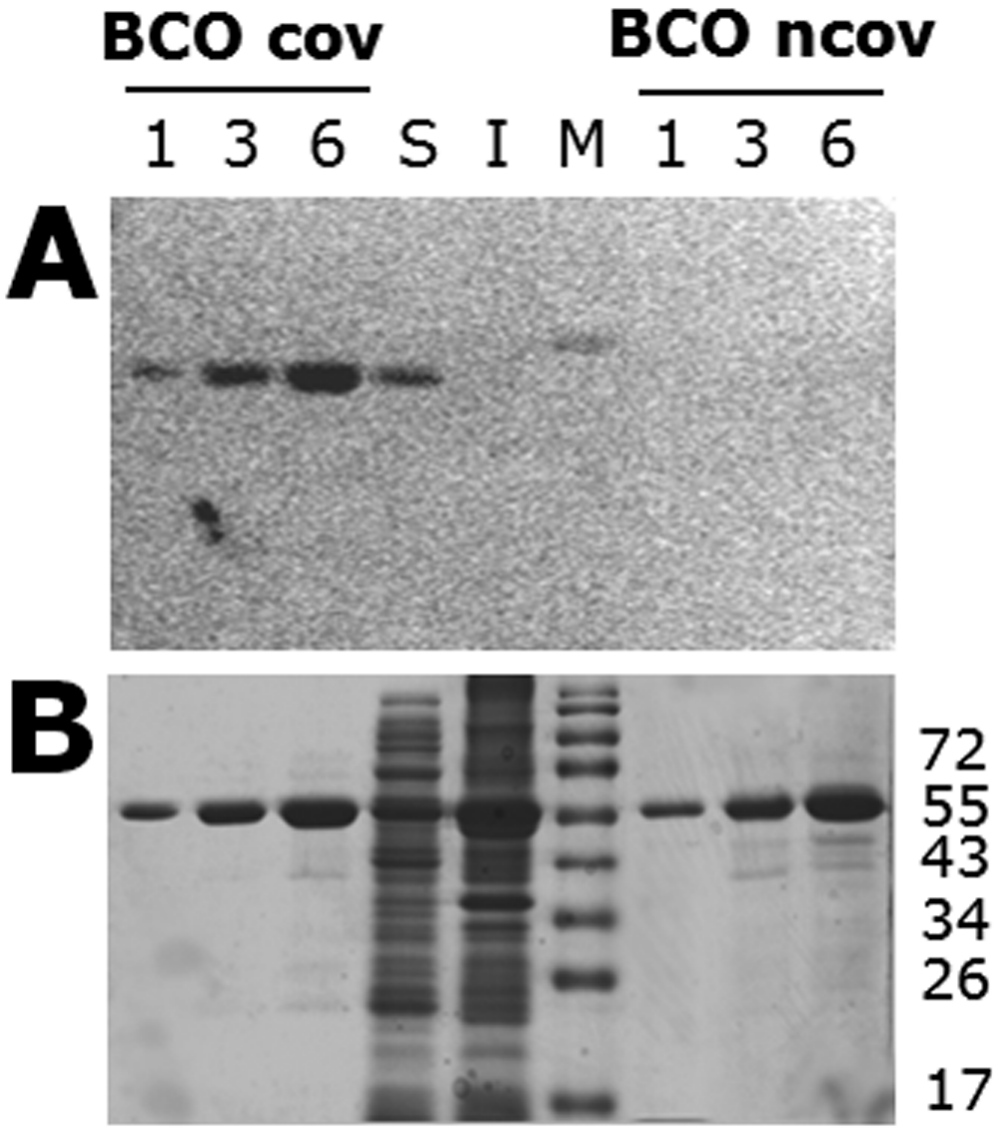
**Determination of the flavinylated *vs. *non-covalent recombinant mBCO by means of SDS-PAGE analysis**. SDS-PAGE Gel was analyzed for flavin fluorescence (panel A) and subsequently stained for total proteins with Coomassie-blue (panel B). Conditions: recombinant *E. coli *cells grown in LB medium, added of 1 mM IPTG at OD_600 nm _= 0.9, then incubated at 25°C and collected after 18 hours. I, Insoluble fraction (pellet after cell disruption and centrifugation); S, soluble fraction (soluble crude extract). An amount of sample corresponding to 0.1 mL of fermentation broth was loaded in each lane. BCOcov and BCOncov: purified flavinylated and non-covalent mBCO forms, respectively; the amount of enzyme loaded are indicate in micrograms. M: molecular weight markers (indicated in kDa on the right side of panel B).

All the above results are based on the measurement of the enzymatic activity in the crude soluble extracts and thus account only for the soluble and catalytically active (i.e., the one containing covalently bound FAD) mBCO. Western-blot analysis of both soluble and insoluble fractions obtained after cells disruption indicate that a significant amount of mBCO is also present in the insoluble fraction (data not shown). Anyway, these analyses do not clarify if the expressed protein is produced or not as covalent flavoprotein. Hence, the relative abundance of the flavinylated *vs*. the non-covalent mBCO form was assessed by the spectral analysis of purified enzyme preparations (the covalent BCO shows absorbance maxima at 368 and 448 nm, while those of the non-covalent BCO are at 389 and 444 nm, with a significant increase in the extinction coefficient at this latter wavelength) and by activity assay (the non-covalent BCO possesses a specific activity 35-fold lower than the covalent one) [6]. In order to assess the effect of the cultivation/expression conditions on the flavinylated *vs. *non-covalent mBCO ratio, we compared the total expression level, as estimated on the basis of Western-blot analysis, with the amount of the covalent form, as determined analyzing flavin fluorescence of SDS-PAGE unstained gels (mBCO form containing the covalently-bound FAD does not lack the cofactor during the electrophoretic separation and thus maintains flavin fluorescence, Figure 3). Under all the tested expression conditions (the ones reported in Tables 2 and 3), recombinant mBCO is present in both the soluble and insoluble fraction, but no flavin fluorescence is observed in the lanes corresponding to the insoluble fractions. These results indicate that when the expressed mBCO accomplishes the folding and the flavinylation process, it becomes a soluble protein, while the non-covalent mBCO remains (at least partially) present as insoluble protein.

**Figure 4:**
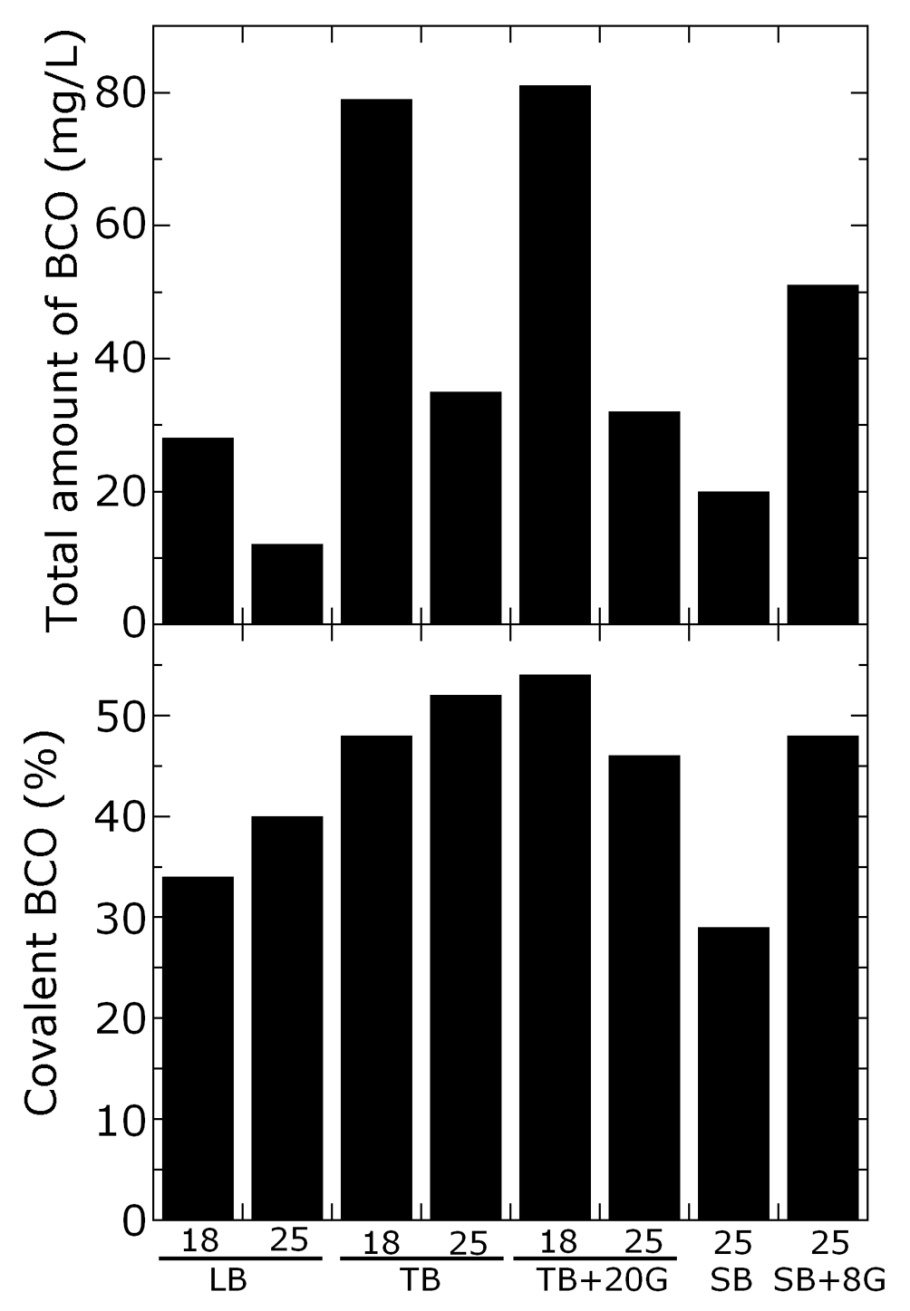
**Purified total soluble mBCO (top panel) and its percentage as flavinylated covalent mBCO (bottom panel)**. Recombinant *E. coli *cells were grown at 37°C, added of 1 mM IPTG at OD_600 nm _= 1.5-2, and harvested after 18 hours at the temperature indicated below each bar. TB+20G: TB medium in the presence of 20 mL/L of glycerol: SB+8G; SB medium in the presence of 8 mL/L of glycerol. Data variability is <10%.

With the aim of understanding the effect of the growth conditions on the flavinylated *vs. *non-covalent mBCO ratio in the soluble protein fraction, the expressed enzyme was purified from the crude extracts by Ni^2+^-affinity chromatography. The highest amount of total soluble mBCO is obtained using TB (with or without additional glycerol) at 18°C (Figure 4, top panel), with a significant increase (about 7-fold) compared to standard conditions (LB medium at 25°C). The percentage of covalent mBCO in respect to the total amount of the produced soluble protein (as estimated by heat denaturation, see Methods) is also higher under these experimental conditions, but it never exceeds the 55% of the soluble mBCO (Figure 4, bottom panel).

## Discussion

Many proteins in nature necessitate the binding of specific cofactors to perform their biological function. These molecules often fold *in vivo *in cellular environments where their cognate cofactors are available, and thus may bind to the appropriate polypeptide before folding. It is generally assumed that cofactors serve as nucleation sites that drive or facilitate the overall folding process since that the search of an unfolded polypeptide for the native state is made easier due to the cofactor's coordination. A number of investigations suggest a post-translational rather than a co-translational process of flavinylation [12]. In this study we investigated the over-expression of a type II-CO from the high G-C gram-positive *Brevibacterium sterolicum*, which is a flavoprotein containing covalently-bound FAD, in the gram-negative *E. coli *as heterologous host. Published results [13-25] only focused on non-covalent (type I) COs expressed in recombinant form either in *E. coli *or *Streptomyces *spp. (see Additional file 1 - Heterologous expression of cholesterol oxidases). Most of them are produced in nature by high G-C gram-positives such as *Streptomyces*, *Rhodococcus *and *Brevibacterium *spp., except those originating from *Burkholderia *and *Chromobacterium *spp. It cannot be excluded that recombinant production of the covalent BCO in *E. coli *may be limited since the folding procedure proceeds in a cellular environment different from that of the natural producer. Nevertheless, optimization of plasmid construct (the one encoding for mature BCO), medium composition (TB added of 20 mL/L of glycerol), growth temperature after induction (21°C) and harvest time (16-18 hours after induction), allowed to increase the amount of mBCO activity expressed in *E. coli *from ~500 up to more that 25000 U/L culture at 2 L fermentor scale and from 0.5 up to more than 7.0 U/mg protein in the crude extract. This expression level compared favourably with previous trials (see Additional file 1 - Heterologous expression of cholesterol oxidases) of non-covalent (type I) COs. Only the expression of *Rhodococcus equi *CO in *Streptomyces lividans *yielded a slightly higher volumetric productivity, although its production time was 5-fold longer [13]. Similarly, longer times were required to obtain a higher specific activity in the crude extract for *Streptomyces spp*. (*ChoA *gene) CO in *E. coli *JM109 [14] (see Additional file 1 - Heterologous expression of cholesterol oxidases).

We also demonstrated that when mBCO is over-expressed in *E. coli*, the flavinylated enzyme form is fully soluble, whereas the non-covalently FAD bound mBCO largely accumulates in insoluble inclusion bodies. The soluble fraction is indeed a mixture of covalent (fully active) and non-covalent mBCO forms: under optimal expression conditions, nearly 55% of the soluble recombinant mBCO is produced as covalently FAD bound form. From the soluble fraction, a >95% homogeneous preparation of flavinylated mBCO can be easily recovered due to the higher thermostability of the covalent form [10]: for example, 3 min of incubation at 45°C in the presence of 0.5 mM cholesterol yields a complete denaturation of the non-covalent form, while the flavinylated mBCO is fully stable. Interestingly, the percentage of covalent mBCO in the soluble crude extract is lower using conditions at which the overall mBCO production is reduced (e.g., in LB medium, see Table 3 and Figure 3), indicating that its production may be not limited by saturation of protein folding machinery or by coenzyme depletion.

## Conclusions

Fully active type II mBCO has been produced in *E. coli *at expression levels comparable with the best ones previously achieved for the biotechnological production of non-covalent (type I) COs (see Additional file 1 - Heterologous expression of cholesterol oxidases). Under these conditions, inclusion bodies contain the insoluble non-covalently bound mBCO, whereas the soluble fraction is a mixture of both the covalently and the non-covalently bound FAD mBCO forms. From the soluble fraction, the flavinylated enzyme can be easily recovered due to its higher thermostability in comparison to the non-covalently bound FAD mBCO form. Taking all together these results, we conclude that the optimization of mBCO over-expression in *E. coli *allows to produce huge amounts of the (fully active) CO containing covalently bound FAD as a novel biotool to be preferentially used in those biotechnological applications (e.g., immobilized enzyme based-process and/or in flow biosensors) where FAD dissociation from type I cholesterol oxidases may reduce the biocatalyst efficiency.

## Methods

### Design, synthesis and cloning of cDNA encoding for different BCO forms

The synthetic cDNA encoding for the mature BCO (mBCO) was designed based on the amino acid sequence reported in the protein 3-D structure (PDB code 1I19) [5], with an additional 9-amino acid N-terminal tag (MSNHHHGHA), which is also present in the commercial BCO (S. Ghisla, personal communication). This additional sequence is not very effective as protein purification tag (the purification yield of commercial BCO on HiTrap chelating column never exceeded 50%) while it appears to play an important role in crystals growth (a successful crystallization of wild-type and mutants of BCO was never achieved using a protein lacking this N-terminal sequence) [2,8]. The full length BCO (fBCO) contains a 52-residue long N-terminal pre-sequence according to the sequence GI:116363, locus P22637 corresponding to the *Brevibacterium sterolicum *CO protein. Notably, the sequence of the original gene is unknown. Both the synthetic cDNA molecules (named mBCO- and fBCO-cDNA, respectively) were synthesized by Medigenomix GmbH (Martinsried, Germany), following optimization of the coding nucleotide sequence for expression in *E. coli*. Both the cDNA molecules were cloned into pET24b(+) expression plasmid (Novagen) using the *Nde*I/*Xho*I restriction sites. The mBCO encoding cDNA was also inserted into a 2.6-kb-shorter version of the pET24b(+) plasmid, obtained by a *Bgl*II/*Tth*111I double digestion followed by treatment with Klenow enzyme and ligation (named pET24Δ plasmid) [11]. Because of the small size, this plasmid was previously demonstrated to increase the transformation and the protein expression yield as compared to the full length one when a large (> 1 kb) exogenous cDNA is cloned [11]. In all the constructs, a 6-His tag (encoded by the plasmid) was added to the C-terminus of the protein. Partial protein sequences of the protein forms obtained by the different cloning strategies, are shown in Figure 1.

### Strain and growth conditions

For protein expression, all plasmids were transferred to the host BL21(DE3)pLysS *E. coli *strain. Starter cultures were prepared using a single colony of *E. coli *carrying the recombinant plasmids in LB medium containing kanamicin (30 μg/mL final concentration) and chloramphenicol (34 μg/mL final concentration) under vigorous shaking at 37°C. The following media were used: M9 (6 g/L Na_2_HPO_4_, 3 g/L KH_2_PO_4_, 1 g/L NH_4_Cl, 0.5 g/L NaCl, 0.05 mM CaCl_2_, 1 mM MgSO_4_, 4 g/L glucose); Luria-Bertani (LB, 10 g/L bacto-tryptone, 5 g/L yeast extract, 5 g/L NaCl); Terrific Broth (TB, 12 g/L bacto-tryptone, 24 g/L yeast extract, 8 mL/L glycerol, 16 mM KH_2_PO_4 _and 54 mM K_2_HPO_4_); TB/glucose (12 g/L bacto-tryptone, 24 g/L yeast extract, 5 g/L glucose, 16 mM KH_2_PO_4 _and 54 mM K_2_HPO_4_); Super Broth (SB, 32 g/L bacto-tryptone, 20 g/L yeast extract, 5 g/L NaCl). When glycerol and/or phosphate salts were added to LB or SB media, the same concentrations as in TB broth were used, unless otherwise stated. Baffled (500 mL) Erlenmeyer flasks containing 80 mL of each medium were inoculated with the starter culture (initial OD_600 nm _= 0.05) and cells were grown at 37°C with shaking (180 rpm) until protein expression was induced.

### Enzyme expression and crude extract preparation

Enzyme expression was induced by the addition of different IPTG concentrations at different phases of the growth curve, as indicated in each case. Crude extracts were prepared by French Press lysis or sonication in 50 mM potassium phosphate buffer, pH 7.5, 1 mM pepstatin, 1 mM phenylmethylsulphonylfluoride (PMSF), 10 μg/mL DNAse. The insoluble fraction of the lysate was removed by centrifugation at 39000 × g for 1 hour at 4°C. For experiments carried out in small volumes (<50 mL), crude extracts were prepared using the CelLytic™ B reagent (Sigma-Aldrich), following the manufacturer's instructions.

### Scaling up to 2 L bioreactor

TB medium added with 20 mL/L glycerol was used as production medium in 2 L working volume P-100 Applikon glass reactor (height 25 cm, diameter 13 cm) equipped with a AD1030 Biocontroller and AD1032 motor. Cultivations in fermentor were carried out at 37°C, 500-700 rpm stirring (corresponding to 1.17-1.64 m/s of tip speed) and 2 L/min aeration rate. Foam production was controlled by the addition of Hodag antifoam through an antifoam sensor. The starter culture was grown overnight in LB medium and diluted up to an initial OD_600 nm _of 0.05. After 1 mM IPTG addition, temperature was shifted down to 21°C and cells were sampled at different times from induction. Three distinct runs were carried out.

### SDS-PAGE Electrophoresis and Western-blot analysis

Proteins from both soluble and insoluble fractions were separated by SDS-PAGE, according to [26]. Unstained polyacrilamide gels were incubated for 1 hour in 10% acetic acid and inspected on an UV-transilluminator. Upon illumination with UV light, the flavinylated BCO was visualized because of the FAD fluorescence [27]. For Western-blot analysis, proteins were transferred electrophoretically onto a nitrocellulose membrane and BCO was detected using anti-His-tag mouse monoclonal antibodies (His-probe, Santa Cruz Biotechnology) and goat anti-mouse IgG HRP conjugated antibodies (Santa Cruz Biotechnology). The recognition was then evidenced by a chemiluminescent method (ECL Plus Western Blotting Detection System, GE Healthcare).

### Determination of enzymatic activity

BCO activity was determined at 25°C using 1 mM cholesterol in 100 mM potassium phosphate at pH 7.5 in the presence of 1% propan-2-ol and 1% Thesit^® ^(v/v final concentrations) by monitoring H_2_O_2 _production using the horseradish peroxidase assay (4 μg/mL, 0.3 mg/mL *o*-dianisidine, Δε_440 _= 13 mM^-1^cm^-1^) as previously described [6,9]. One BCO unit corresponds to the amount of enzyme that produces one μmol of hydrogen peroxide per minute, at 25°C.

### Purification of recombinant mBCO and determination of covalent vs. non-covalent mBCO ratio

Recombinant mBCO containing a C-terminal His-tag was purified using Ni^2+^-affinity chromatography (HiTrap Chelating HP columns equilibrated with 50 mM potassium phosphate, pH 7.5, 1 M NaCl) [10]. Elution of bound enzyme was performed with a linear gradient of imidazole (0-0.5 M) in 50 mM potassium phosphate, pH 7.5, 0.5 M NaCl: recombinant mBCO eluted at about 0.25 M imidazole. Imidazole was then removed by gel-permeation chromatography on a PD10 column (GE Healthcare) equilibrated with 100 mM potassium phosphate buffer, pH 7.5, or by extensive dialysis against the same buffer.

The total amount of purified mBCO was determined spectrophotometrically using isosbestic points between absorption spectra of covalent and non-covalent forms of BCO at 372 nm (ε = 8.97 mM^-1^cm^-1^) and at 481 nm (ε = 9.26 mM^-1^cm^-1^) [6]. The difference between the concentrations determined at these two wavelengths was in all cases lower than 5%. The amount of the non-covalent mBCO was determined following heat denaturation (5 min at 100°C) and centrifugation (10 min at 13000 rpm in a Heraeus Biofuge, 4°C) to remove the precipitated protein. Upon denaturation, non-covalent mBCO releases FAD, that remains in the surnatant: its concentration (corresponding to the non-covalent mBCO) was determined spectrophotometrically using ε_450 nm _= 11.3 mM^-1^cm^-1^.

## Competing interests

The authors declare that they have no competing interests.

## Authors' contributions

FV carried out the expression experiments, participated to the molecular biology work and helped to draft the manuscript, L. Pollegioni conceived the study, participated in its design and coordination and assembled the manuscript, GM carried out the molecular cloning and the bioinformatics analysis, FM designed the microbiological and fermentation experiments and helped to write the manuscript, L. Piubelli carried out the biochemical enzymatic assays, set-up the electrophoretic method to quantify the expression of covalent and non-covalent mBCO, designed the expression experiments and helped to write the manuscript, LF performed the experiments at the fermentor scale. All the authors read and approved the final manuscript.

## Supplementary Material

Additional file 1 **Production of recombinant cholesterol oxidases (type I) in different heterologous hosts**. summary of the expression systems for cholesterol oxidase from different sources available up to now. Click here for file
