# Supplementary material for: Production of recombinant cholesterol oxidase containing covalently bound FAD in Escherichia coli
Source: BMC Biotechnol. 2010 Apr 21;10:33. doi: 10.1186/1472-6750-10-33 (PMC2890692; doi:10.1186/1472-6750-10-33)
Supplement: Additional file 1 — Production of recombinant cholesterol oxidases (type I) in different heterologous hosts. summary of the expression systems for cholesterol oxidase from different sources available up to now. [file 1472-6750-10-33-S1.PDF]

Production of recombinant cholesterol oxidases (type I) in different heterologous hosts.

Source microorganism	Host (plasmid) [promoter]	Notes	Expression level (U/L)	Expression level (U/mg$_{protein}$)	Reference (see text)
Rhodococcus equi (uncorrectly classified as Brevibacterium sterolicum ATCC 21387)	*E. coli* MM294 (pnH10) [P*lac*]	- non covalent<br>- growth at 30 °C, 16 hours, in LB medium, no induction<br>- MW = 46.5 kDa	585[a] (79 U/g cell)	0.58[a] (pure 55.2 U/mg)	[13]
	E. coli BL21(DE3)pLysS (pBCO4) [PT7*lac*]	- modification of the codon usage of the N-terminal 21 residues<br>- growth in 2xYT medium, induction with 1 mM IPTG at mid-log phase (OD_{600nm} = 0.6), growth for additional 10 hours at 37 °C<br>- MW = 55.4 kDa	2180[a]	2.2[a] (pure 45 U/mg)	[14]
	E. coli JM105 (pCHS43) [P*lac*Z]	- growth in LB medium, induction with 1 mM IPTG<br>- MW ~ 54 kDa	360	n.d	[15]
	Streptomyces lividans TK23 (pCHS31) [P*mel*]	- growth at 30 °C in SK2 medium with with 20 µg/mL thiopeptin for 120 hours	29500[b] (600 mg/L)	1.8[b]	
Rhodococcus sp. PCTT 1663	*E. coli* BL21(DE3)pLysS (pET23a) [PT7]	- growth in LB medium, induction with 0.4 mM IPTG, growth at 30 °C for 4 hours<br>- MW = 55 kDa	2150[a]	2.2[a] (pure 37.6 U/mg)	[16]
Brevibacterium sp. CCTCC M201008 (98% identity with ATCC 21387)	*E. coli* BL21 – Codon plus (DE3)-RP (pET28) [PT7]	- MW ~ 60 kDa<br>- growth in LB medium, induction with 0.8 mM IPTG, growth at 23 °C for 3 hours, ±50 µM riboflavin<br>- the specific activity in the crude extract increases at decreasing growth temperature	n.d.	3.7[a]	[17]

Production of recombinant cholesterol oxidases (type I) in different heterologous hosts (continued).

Source microorganism	Host (plasmid) [promoter]	Notes	Expression level (U/L)	Expression level (U/$mg_{protein}$)	Reference (see text)
Streptomyces coelicola	*Bifidobacterium longum* (pBES16PR) [P16S RNA]	- maximal production after 9 hours	n.d.	0.65[a]	[18]
Streptomyces spp. (*cho*A)	*E. coli* HB101 (pCHOA) [P*lac*]	- growth at 37 °C in LB medium; induction with 0.4 mM IPTG; cells collected after 16 hours	n.d.	0.01[a]	[19]
	Propionibacterium/E. coli (pWK7) [P*acc*]		n.d.	0.004[a]	[20]
	(pPK705CO8) [P*acc*]	- growth at 32 °C in NLB medium	n.d.	4.3[a]	
	E. coli JM109 (pPK705CO8) [P8]	- growth for 3 days	n.d.	9.7[a]	
	E. coli JM109 (pCO117) [P*tac*]	- growth in LB medium, induction with 1 mM IPTG, cells collected after 8 hours<br>- MW ~ 57 kDa	n.d.	1.5 (pure 23.5 U/mg)	[21]
	Streptomyces lividans (pIJ702) (pCO3)	- growth in GMP medium, at 28 °C up to 8 days<br>- up to 40% of expressed protein is extracellular	~ 7500[a] intracellular<br>~ 3800[a] extracellular	n.d.	[22]
	Streptomyces lividans 1326 (pCO100A) [P*lac*]	- 90% of recombinant CO is secreted<br>- maximal production after 5 days of growth in YEME medium at 28 °C<br>- contain ~180 plasmid copies/cell	4300	n.d.	[23]

Production of recombinant cholesterol oxidases (type I) in different heterologous hosts (continued).

Source microorganism	Host (plasmid) [promoter]	Notes	Expression level (U/L)	Expression level ($U/mg_{protein}$)	Reference (see text)
Burkholderia cepacia ST-200 (uncorrectly identified as *Pseudomonas* sp. ST-200)	*E. coli* DH5α (pCOX4) [P*lac*]	- growth in LBG medium for 24 hours<br>- 73% of recombinant CO is periplasmic<br>- proteolysis of signal peptide upon excretion and secretion (MW ~ 59.5 kDa)	67	0.2[c] (pure 16.9 U/mg)	[24]
Chromobacterium DS-1	*E. coli* Rosetta (pETCOX) [pT7*lac*]	- growth in LB medium, induction with 1 mM IPTG at an OD_{600} = 0.5, cells collected after 12 hours at 30 °C	1850	2.3[c]	[25]

[a]The activity was determined at 37 °C.

[b]The specific activity of the purified enzyme is ~6-fold higher than in all published data (288 *vs.* ~50 U/mg protein): the reported values have been corrected thoroughly.

[c]The activity was determined at 30 °C.

n.d., not determined.
